# Healthcare Expenditure and Health System Efficiency in 25 European Countries: A Multidimensional Data Envelopment Analysis with Bootstrap Correction and Second-Stage Regression

**DOI:** 10.3390/epidemiologia7040092

**Published:** 2026-07-02

**Authors:** Antonio Pinto, Flavia Pennisi, Carlo Signorelli

**Affiliations:** 1 Faculty of Medicine, University Vita-Salute San Raffaele, 20132 Milan, Italy; pinto.antonio@hsr.it (A.P.); signorelli.carlo@hsr.it (C.S.); 2 PhD National Programme in One Health Approaches to Infectious Diseases and Life Science Research, Department of Public Health, Experimental and Forensic Medicine, University of Pavia, 27100 Pavia, Italy

**Keywords:** data envelopment analysis, health system efficiency, healthcare expenditure, Europe, public health, bootstrap DEA, Simar–Wilson

## Abstract

Background: European health systems face growing pressure from population ageing, post-pandemic service backlogs, and fiscal constraints. Yet substantial cross-country differences in health outcomes persist despite comparable levels of healthcare expenditure. This study evaluated the relative efficiency of European health systems using a multidimensional framework that integrates expenditure, prevention, and population health outcomes. Methods: A cross-sectional analysis was conducted on 25 European countries using 2022 data or the nearest available year. An output-oriented constant returns to scale Data Envelopment Analysis (DEA) model was estimated with two inputs, public and private healthcare expenditure per capita, and five outputs, life expectancy at birth, inverse infant mortality, healthy life years at birth, breast cancer screening coverage, and poliomyelitis vaccination coverage. A robustness specification added physician density as an additional input. Bootstrap bias correction with 1000 replications was applied to the baseline model. A second-stage Simar–Wilson truncated regression with 2000 bootstrap replications examined the association between inefficiency and selected contextual variables, including GDP per capita, population ageing, obesity prevalence, and tobacco use prevalence. Results: In the baseline DEA model, 8 of 25 countries were located on the technical efficiency frontier (Croatia, Czechia, Estonia, Greece, Hungary, Latvia, Lithuania, and Poland; output-oriented DEA inefficiency score = 1.000 for each country), while inefficiency scores among the remaining countries ranged from 1.042 to 2.617. The highest inefficiency scores were observed for Germany (2.617), Austria (2.283), Belgium (2.230), Ireland (2.219), and France (2.167). When physician density was added as an additional input, 12 countries were located on the estimated frontier. Bootstrap correction of the baseline model increased the estimated output-oriented inefficiency scores, with bias-corrected values ranging from 1.100 to 2.941. In the second-stage analysis, higher log GDP per capita was positively associated with bias-corrected inefficiency (coefficient 1.993; 95% bootstrap CI 0.219 to 4.197), whereas population ageing, adult obesity prevalence, and tobacco use prevalence were not statistically associated with bias-corrected inefficiency. Conclusions: In this cross-sectional sample of 25 European countries, higher healthcare expenditure was not consistently associated with frontier performance when health outcomes and preventive coverage were considered jointly. The results were sensitive to the inclusion of physician density and to bootstrap correction, supporting the interpretation of Data Envelopment Analysis as an exploratory benchmarking tool rather than a definitive ranking of health systems. These findings highlight the importance of assessing how financial and workforce resources are converted into measurable health and prevention-related outputs.

## 1. Introduction

European health systems are facing increasing pressures driven by population ageing [1,2,3] post-pandemic backlogs [4,5], and tightening fiscal constraints [6]. At the same time, substantial cross-country differences persist in health outcomes despite comparable levels of expenditure, suggesting that performance depends not only on how much is spent, but also on how efficiently resources are transformed into health [7].

In this context, analytical approaches capable of disentangling expenditure levels from productive efficiency are essential. Data Envelopment Analysis (DEA) provides a non-parametric framework for benchmarking health systems against a best-practice frontier, allowing for the identification of countries that achieve relatively high health outcomes given their available resources [8,9]. Previous studies have applied DEA to European health systems, typically relying on a single outcome such as life expectancy or on a very limited set of indicators [10,11]. Focusing on one outcome alone risks overlooking important dimensions of system performance, particularly prevention and broader population health. Moreover, standard DEA models may be sensitive to sampling variability and may assign negligible weights to clinically relevant indicators, limiting interpretability for health policy [12]. 

Although DEA has been widely used in comparative health system research, prior cross-country applications have often relied on relatively narrow output sets and have not always explicitly addressed statistical uncertainty in frontier estimation. In addition, prevention-related indicators have generally received less attention than aggregate mortality- or longevity-based measures, despite their relevance for public health-oriented assessments of health system performance.

To address these limitations, this study evaluates the efficiency of European health systems using a multidimensional set of five outputs that capture both downstream health outcomes associated with healthcare expenditure and upstream preventive performance, including screening coverage and vaccination uptake [13,14,15,16]. A bootstrap DEA approach is employed to account for statistical uncertainty, followed by a second-stage truncated regression to explore the association between efficiency and selected socioeconomic and lifestyle factors. Rather than providing a definitive ranking of national health systems, the analysis is intended as a multidimensional and uncertainty-aware benchmarking exercise. By adopting this integrated framework, the analysis provides a comprehensive assessment of health system value-for-money across Europe and offers empirical insights into the extent to which efficiency differentials may reflect broader contextual characteristics rather than health spending alone. In this way, this study contributes to the literature by combining a broader output specification with bootstrap bias correction and second-stage analysis in a recent European sample.

## 2. Materials and Methods

### 2.1. Study Design, Sample and Data Sources

This cross-sectional study assessed the relative efficiency of national health systems across 25 European countries in 2022. The final sample was defined by complete-case availability across all variables included in the main DEA model, the robustness specification, and the second-stage analysis. Most variables referred to 2022; physician density, included only in the robustness analysis, referred to 2021 as the most recent harmonized year available. 

The analytical dataset combined expenditure, health outcome, prevention, and health system resource indicators derived from publicly available international databases. Public healthcare expenditure per capita and private healthcare expenditure per capita, both expressed in purchasing power parity-adjusted current international dollars, were obtained from the World Bank World Development Indicators. Life expectancy at birth was obtained from the United Nations Data Portal [17], infant mortality from the Organisation for Economic Co-operation and Development (OECD) Data Explorer [18], healthy life years at birth from Eurostat [19], and poliomyelitis vaccination coverage from the World Health Organization (WHO) immunization database [20]. Healthy life years referred to 2022 for all countries except the United Kingdom, for which 2018 was the most recent available year in the same source. Poliomyelitis vaccination coverage was defined as official coverage for the third dose. Physician density from OECD [21], measured as doctors per 1000 population, was used in the robustness analysis. Breast cancer screening coverage was primarily obtained from the OECD Data Explorer; where directly comparable 2022 values were not available in the comparative extract, country-specific OECD [22]/European Commission Cancer Country Profiles were used to complete the dataset, as in the cases of Spain [23] and Portugal [24].

In the second-stage analysis, the explanatory variables were GDP per capita in purchasing power standards, obtained from Eurostat [25], the proportion of the population aged 65 years and over, obtained from the World Bank [26], adult obesity prevalence among individuals aged 18 years and over, obtained from the WHO [27], and current tobacco use prevalence, also obtained from the WHO [28]. GDP per capita was log-transformed before modelling.

Variable selection was guided by the objective of capturing both health system resource use and multidimensional performance using indicators that were internationally comparable and available for the study sample. Public and private healthcare expenditure per capita were selected as financial inputs because they represent the main monetary resources mobilized by health systems, while retaining them separately preserved information on differences in financing structure. Life expectancy at birth was included as a broad measure of survival, infant mortality as an indicator of early-life health and health system responsiveness, and healthy life years as a measure of longevity adjusted for functional health. Breast cancer screening coverage and poliomyelitis vaccination coverage were included to capture secondary and primary prevention, respectively. Physician density was added in the robustness specification because workforce availability is a key dimension of health system capacity. The second-stage covariates were selected to represent macroeconomic context, demographic pressure, and major behavioural risk factors that may influence health system performance but are not direct outputs of healthcare production [29].

### 2.2. Variable Construction

To ensure directional consistency across outputs, infant mortality was transformed as its inverse prior to analysis, so that higher values uniformly represented better performance. Public and private healthcare expenditure were retained as separate inputs rather than aggregated into a single spending variable, in order to preserve information on different financing components and to assess health system performance in relation to the observed configuration of expenditure.

The selected outputs were not intended to represent a single homogeneous construct. Rather, they were chosen to capture complementary dimensions of health system performance, including long-term population health outcomes and prevention-related service coverage. This broader specification was adopted to avoid an exclusively mortality-based representation of performance, while acknowledging that these indicators differ in their causal proximity to healthcare delivery and may therefore reflect partially distinct production processes.

Accordingly, the mixed output setting was interpreted as a multidimensional benchmarking framework rather than as a single homogeneous health production function. Its validity was assessed conceptually by ensuring that all outputs were desirable indicators, measured for the same country-level units, internationally comparable, and relevant to health system performance, while explicitly acknowledging their different causal distance from healthcare delivery.

### 2.3. DEA Model Specification

Efficiency was estimated using DEA [30], a non-parametric linear programming technique that constructs an empirical best-practice frontier and evaluates each country relative to that frontier.

For each country o, with input vector $x_{o}$ and output vector $y_{o}$, the output-oriented CRS DEA model was specified as follows:

$$\underset{\phi_{o},\lambda }{max\;}\phi_{o}$$

subject to the following:

$$\sum_{j=1}^{n}\lambda_{j}y_{rj}\geq \phi_{o}y_{ro},r=1,\ldots ,s$$

$$\sum_{j=1}^{n}\lambda_{j}x_{ij}\leq x_{io},i=1,\ldots ,m$$

$$\lambda_{j}\geq 0,j=1,\ldots ,n$$

where n is the number of countries, m is the number of inputs, s is the number of outputs, $\lambda_{j}$ are intensity weights, and $\phi_{o}$ is the output-oriented radial expansion factor for country o. Under this convention, $\phi_{o}=1$ indicates that the country is located on the estimated frontier, whereas $\phi_{o}>1$ indicates the proportional expansion in outputs required for country o to reach the frontier, conditional on its observed inputs.

An output-oriented specification was adopted because the objective of the study was to assess the extent to which countries were able to achieve higher levels of health and prevention performance given their observed resource use. Under this framework, inefficiency reflects the proportional shortfall in outputs relative to the frontier, conditional on the level of inputs. 

The baseline DEA model was estimated under constant returns to scale (CRS) [31]. The CRS specification was adopted to focus on overall technical efficiency under the assumption that proportional differences in inputs were associated with proportional differences in attainable outputs. This choice was also supported by the use of per capita or otherwise standardized indicators, which reduced, although did not eliminate, the influence of absolute country size. However, this assumption remains strong in cross-country health system comparisons and should be interpreted as a modelling choice rather than an empirically verified property of the underlying production technology. The baseline model included two financial inputs, namely public healthcare expenditure per capita and private healthcare expenditure per capita, and five outputs: life expectancy at birth, inverse infant mortality, healthy life years at birth, breast cancer screening coverage, and poliomyelitis vaccination coverage. This output set was selected to reflect both health status and preventive/public health performance, rather than relying on a single mortality-based indicator. Public and private expenditure were modelled separately because they represent distinct financing components of health system resource use and may reflect different institutional configurations. Retaining them as separate inputs allowed the analysis to preserve information on the observed expenditure mix rather than imposing an aggregated spending measure a priori. At the same time, this choice should not be interpreted as evidence that these components operate through fully separable production mechanisms. 

A secondary DEA specification was estimated as a robustness analysis by adding physician density as an additional input. This model was used to explore whether part of the inefficiency observed in the expenditure-only model might reflect differences in health system capacity. Alternative variable returns to scale specifications were explored during model development but were not retained as the main analytical approach because they provided limited discriminatory power in this dataset.

### 2.4. Bootstrap DEA

Because conventional DEA estimates are deterministic and sensitive to sampling variation, a bootstrap procedure following the Simar–Wilson approach [32] was applied to the baseline model. Bias-corrected inefficiency estimates and percentile-based 95% confidence intervals were obtained using 1000 bootstrap replications. The number of bootstrap replications was selected to balance computational feasibility and precision of the bias-corrected estimates. As a stability check, the bootstrap DEA procedure was repeated using 500, 1000, and 2000 replications.

In the output-oriented formulation used in this study, the reported DEA score corresponds to the radial output expansion factor, denoted as ϕ. Therefore, scores below 1 are not possible by construction. A value of ϕ=1.000 indicates that a country is located on the estimated frontier, whereas values greater than 1 indicate the proportional output expansion required to reach the frontier, conditional on observed inputs. For example, ϕ=1.042 indicates that outputs would need to be radially expanded by approximately 4.2% to reach the frontier. The reciprocal value 1/ϕ corresponds to the conventional bounded efficiency scale ranging from 0 to 1, but the present study reports ϕ because it is the direct output-oriented DEA expansion factor used in the analysis. The bootstrap analysis was included to correct for bias in frontier estimates and to quantify uncertainty around country-level inefficiency scores.

### 2.5. Second-Stage Analysis

To examine whether between-country differences in inefficiency were associated with broader contextual characteristics, a second-stage regression analysis was performed using the bias-corrected DEA inefficiency scores as the dependent variable. Following the Simar–Wilson framework, a truncated regression model with left truncation at 1 was estimated, consistent with the bounded support of output-oriented DEA inefficiency scores. Statistical inference was based on a second bootstrap procedure with 2000 replications. The number of second-stage bootstrap replications was selected to improve the precision of percentile-based confidence intervals. Stability was assessed by repeating the second-stage bootstrap using 1000, 2000, and 5000 replications.

The second-stage model was specified as follosw:

$${\hat{\phi }}_{o}^{BC}=\beta_{0}+\beta_{1}log(GDPpc_{o})+\beta_{2}Age65_{o}+\beta_{3}Obesity_{o}+\beta_{4}Tobacco_{o}+\varepsilon_{o}$$

with left truncation at 1, consistent with the support of the dependent variable:

$${\hat{\phi }}_{o}^{BC}>1$$

where ${\hat{\phi }}_{o}^{BC}$ denotes the bootstrap bias-corrected output-oriented DEA inefficiency score for country o, $log(GDPpc_{o})$ is log GDP per capita in purchasing power standards, $Age65_{o}$ is the proportion of the population aged 65 years and over, $Obesity_{o}$ is adult obesity prevalence, and $Tobacco_{o}$ is current tobacco use prevalence. Positive coefficients indicate higher bias-corrected inefficiency, whereas negative coefficients indicate lower bias-corrected inefficiency. 

Nested model specifications were also estimated as sensitivity analyses by progressively adding covariates in order to assess the robustness of the observed associations.

### 2.6. Software

All analyses were performed using R, version 4.4.2 (R Foundation for Statistical Computing, Vienna, Austria). DEA and bootstrap DEA procedures were implemented using the Benchmarking package, version 0.33, and second-stage truncated regression was estimated using the truncreg package, version 0.2-5.

## 3. Results

### 3.1. Baseline DEA Results

The baseline output-oriented CRS DEA model, based on two expenditure inputs and five health outputs, identified 8 of the 25 countries as technically efficient. These countries were Croatia, Czechia, Estonia, Greece, Hungary, Latvia, Lithuania, and Poland, all with an efficiency score equal to 1.00. The remaining 17 countries had output-oriented inefficiency scores greater than 1, indicating that they were located below the estimated frontier. These values ranged from 1.042 to 2.617, corresponding to a required radial output expansion of approximately 4.2% to 161.7% relative to the CRS frontier, conditional on observed inputs. 

Full country-specific results are reported in Table 1.

### 3.2. Robustness Analysis Including Physician Density

In the robustness specification, physician density was added as a third input alongside public and private healthcare expenditure. Under this specification, 12 countries were identified as technically efficient: Croatia, Czechia, Estonia, Finland, France, Greece, Hungary, Latvia, Lithuania, Luxembourg, Poland, and Slovenia.

Relative to the baseline model, several countries moved closer to the frontier. Four countries that were not technically efficient in the baseline model, namely Finland, France, Luxembourg, and Slovenia, were classified as technically efficient in the physician-adjusted model. Among countries that remained inefficient, the scores ranged from 1.007 for the United Kingdom to 1.598 for Austria. Portugal was unchanged at 1.230. Table 2 reports the full results of the robustness analysis.

### 3.3. Bootstrap DEA Results

Bootstrap correction of the baseline DEA model yielded systematically higher inefficiency estimates than the original scores. Bias-corrected values ranged from 1.100 (95% CI 1.016–1.200) for Lithuania to 2.941 (95% CI 2.652–3.299) for Germany. The countries with the highest bias-corrected inefficiency scores were Germany, Austria, Belgium, Ireland, and France, mirroring the ranking observed in the original DEA model.

For countries originally located on the frontier, the bootstrap procedure produced bias-corrected scores above 1, indicating that apparent full efficiency in the deterministic DEA framework was sensitive to sampling variation. Accordingly, countries located on the deterministic frontier should be interpreted as near-frontier reference points generated by the baseline model rather than as unequivocal benchmarks. For example, Lithuania, Poland, Czechia, and Hungary had bias-corrected scores between 1.100 and 1.173 despite original scores equal to 1.000. Confidence intervals were wider among countries with higher corrected inefficiency scores, particularly for Germany, Austria, Belgium, Ireland, and the United Kingdom. Full bootstrap results are shown in Table 3.

### 3.4. Second-Stage Simar–Wilson Regression

The second-stage truncated regression was performed using bootstrap bias-corrected DEA inefficiency scores as the dependent variable. Among the explanatory variables considered, only log GDP per capita was associated with inefficiency in the fully adjusted model. The estimated coefficient for log GDP was 1.993, with a 95% bootstrap confidence interval ranging from 0.219 to 4.197.

By contrast, the proportion of the population aged 65 years and over, adult obesity prevalence, and tobacco use prevalence were not statistically associated with bias-corrected inefficiency scores. The coefficient estimates were 0.121 for population aged 65 years and over, −0.043 for obesity prevalence, and −0.008 for tobacco use prevalence. The results of the second-stage model are presented in Table 4.

### 3.5. Stability Checks for Bootstrap Replications

Stability checks showed that the bootstrap DEA results were robust to the number of replications. Compared with the main 1000-replication specification, the 500- and 2000-replication models showed near-identical rankings, with Spearman rank correlations of 0.999 and 1.000, respectively. Pearson correlations for the bias-corrected scores were also very high, equal to 0.99997 and 0.999998, respectively. Mean absolute differences in bias-corrected scores were small, equal to 0.0029 for 500 replications and 0.0009 for 2000 replications. The maximum rank difference was one position for the 500-replication model and zero positions for the 2000-replication model. The second-stage bootstrap results were also stable across 1000, 2000, and 5000 replications: log GDP per capita remained positively associated with bias-corrected inefficiency, whereas population ageing, obesity prevalence, and tobacco use prevalence remained non-significant.

## 4. Discussion

### 4.1. Summary of Results

This study assessed the relative efficiency of 25 European health systems using an output-oriented DEA framework with two financial inputs and a multidimensional set of five outputs capturing both health status and preventive performance. Several findings deserve emphasis. First, the baseline model showed marked heterogeneity across countries, with only 8 of 25 systems located on the efficiency frontier and wide variation in inefficiency scores. Second, the robustness analysis showed that part of the observed inefficiency was attenuated when physician density was added to the model, suggesting that expenditure alone does not fully capture the productive capacity of a health system. Third, bootstrap correction increased inefficiency estimates across countries, confirming that deterministic DEA may overstate apparent efficiency when sampling variation is ignored. Finally, in the second-stage analysis, only GDP per capita was associated with inefficiency in the fully adjusted model, whereas the proportion of older adults, obesity prevalence, and tobacco use were not statistically associated with the bias-corrected scores.

### 4.2. Interpretation of Main Findings

Taken together, these results reinforce a central message in the health system efficiency literature: higher expenditure does not automatically translate into better population health performance [33]. Earlier comparative studies of OECD and European countries reached similar conclusions, showing that some countries achieve favourable outcomes with relatively modest resources, whereas others spend substantially more without proportionate gains [34,35]. Retzlaff-Roberts and colleagues [36] argued that “more is not necessarily better” in the allocation of healthcare resources, while later work has likewise shown that wide cross-country variation persists even among systems operating at comparable spending levels. Our findings are consistent with this interpretation and extend it by showing that the mismatch between spending and performance remains evident even when performance is defined more broadly than life expectancy alone.

An important strength of the present study is precisely this broader conception of output. Much of the earlier DEA literature has relied on a small set of aggregate indicators, often limited to life expectancy, healthy life expectancy, and infant mortality [37,38]. While these remain important, they do not fully reflect preventive and public health dimensions of system performance. By incorporating breast cancer screening and poliomyelitis vaccination coverage alongside survival-related outcomes, the present analysis captures a wider representation of what health systems are expected to deliver. From a public health perspective, this is highly relevant. Under the present multidimensional specification, a country located near the frontier for downstream outcomes but weaker in prevention would not necessarily represent balanced relative performance across all selected outputs [39]. Conversely, preventive performance may partly explain why some systems achieve good outcomes despite lower expenditure [40].

At the same time, this broader output specification introduces an important interpretive constraint. The selected indicators do not occupy the same conceptual level within the health production process: life expectancy and healthy life years reflect long-term aggregate outcomes, whereas screening and vaccination coverage are more proximal indicators of preventive service delivery. Accordingly, the estimated frontier should be interpreted as a multidimensional benchmarking construct rather than as the output of a single fully homogeneous production function.

The robustness analysis including physician density offers an additional insight. The movement of several countries closer to the frontier after adding doctors per 1000 population suggests that part of what appears to be “expenditure inefficiency” in the baseline model may actually reflect differences in structural capacity. In other words, some systems may not be inefficient simply because they spend more, but because expenditure is translated through a different staffing configuration and service-production structure. This interpretation aligns with the broader literature emphasizing that effective staffing models, appropriate asset use, and the organization of service delivery are integral components of health system productivity, beyond financial inputs alone [41,42,43]. From a health policy standpoint, this is important because it cautions against interpreting expenditure levels in isolation. A country may have high expenditure and still show a higher model-specific inefficiency score if resources are absorbed by fragmented service delivery, workforce shortages in key sectors, suboptimal skill mix, or weak integration between primary, preventive, and specialized care [44,45]. However, this finding should remain narrowly interpreted. Because physician density is conceptually related to expenditure and only one additional capacity indicator was introduced, the robustness model cannot identify the specific organizational mechanisms underlying cross-country differences in inefficiency. It only shows that country classifications were sensitive to the inclusion of a workforce-related input. Therefore, the observed changes should not be interpreted as evidence of a specific mechanism, such as physician shortage, geographical maldistribution, suboptimal skill mix, or low productivity. Distinguishing among these mechanisms would require subnational or service-level workforce, utilization, and productivity data that were not available in the present cross-country dataset.

The bootstrap results also deserve substantive interpretation. The upward shift in inefficiency scores after bias correction indicates that some countries classified as fully efficient under conventional DEA are better interpreted as near-efficient rather than unequivocally efficient once statistical uncertainty is considered. This is fully consistent with previous methodological studies showing that standard DEA may overestimate efficiency and that bootstrap procedures improve the credibility of cross-country comparisons [46,47]. In practice, this means that efficiency frontiers should not be interpreted too rigidly. In a policy context, DEA is best viewed as a benchmarking tool rather than as an absolute judgement of health system quality. Accordingly, countries located on the deterministic frontier should be used as model-based reference points for comparative learning, not as definitive policy benchmarks. The recent literature has stressed exactly this point: DEA is highly informative for comparative assessment, but its outputs should support structured reflection and policy learning, not simplistic judgement [48].

The second-stage findings require cautious interpretation. The positive association between log GDP per capita and inefficiency may appear counterintuitive at first glance, because wealthier countries generally have more resources, stronger infrastructure, and better health outcomes [49,50]. However, in relative efficiency terms, higher-income systems may also face higher cost structures, greater technological intensity, stronger demand pressures, and diminishing returns to additional expenditure [51]. Previous work [52] has noted that richer countries may spend substantially more without obtaining proportionally higher marginal gains in outcomes. In this sense, the GDP finding may reflect the fact that affluent systems are not judged against an absolute outcome threshold, but against what is attainable given their resource levels. Put differently, richer countries may achieve good outcomes, yet still appear relatively inefficient if these outcomes fall short of what other systems obtain with fewer resources. This association should therefore be regarded as hypothesis-generating rather than explanatory in a strong sense. Given the small sample and the model-relative nature of DEA inefficiency scores, the regression results are better interpreted as exploratory evidence of patterning than as evidence of a stable structural relationship between national income and health system inefficiency.

By contrast, the lack of significant associations for population ageing, obesity, and tobacco use should not be interpreted as evidence that these factors are unimportant for health system performance or as contradicting the broader literature on population health determinants. The stability checks further indicated that these null associations were not driven by the selected number of second-stage bootstrap replications, as the same substantive inference was observed using 1000, 2000, and 5000 replications. Instead, these findings indicate a lack of detectable association with model-specific relative inefficiency scores in this small ecological sample. This may reflect the limitations of ecological cross-country modelling, limited statistical power, the aggregate nature of the indicators, and the possibility that these variables operate over long time horizons, interact with policy and social context, or affect specific disease burdens more strongly than aggregate system efficiency scores [53]. The broader literature repeatedly emphasizes that external determinants of health complicate efficiency measurement and may not be fully captured by aggregate national indicators [54,55]. Their non-significance in the present study therefore argues more for interpretive caution than for dismissal of their relevance.

### 4.3. Implications for Policies and Public Health Practices

The results support a shift in policy attention from expenditure volume alone to expenditure conversion, that is, how effectively financial resources are translated into survival, healthy longevity, and preventive coverage. For European health systems under fiscal pressure, this is a crucial distinction. Increasing expenditure may be necessary in some settings, but it is unlikely to be sufficient unless accompanied by reforms in governance, service integration, workforce planning, and prevention [56]. The identification of countries closer to or farther from the estimated frontier provides a basis for structured benchmarking. Comparative learning should not assume the direct transferability of institutional arrangements, but it can still highlight policy areas in which similar or better outcomes are being achieved at lower resource intensity. This benchmarking logic is a core reason why international efficiency comparisons remain valuable for policymakers [57]. Again, the physician-adjusted model suggests that workforce capacity deserves strategic priority. In practical terms, improving value-for-money in health systems may depend as much on strengthening workforce availability and skill mix as on increasing budgetary allocations [58,59].

### 4.4. Limitations

First, this study is based on aggregate national data and therefore cannot capture within-country heterogeneity, regional inequalities, or differences in access and quality across population groups. Second, differences in health system architecture, including financing models, decentralization, gatekeeping arrangements, provider payment systems, benefit design, and the public–private mix of service delivery, were not directly modelled as categorical confounders. These institutional features may influence both resource use and the conversion of expenditure into health and prevention-related outputs. Although the analysis partially addressed this heterogeneity by modelling public and private expenditure separately and by testing physician density in the robustness specification, residual institutional confounding may remain. Future studies using larger panel datasets could more explicitly examine health system typologies or governance characteristics. Third, DEA remains sensitive to variable selection and sample composition. Although the chosen output set was broader than in many previous studies, it combined distal population health outcomes, such as life expectancy and healthy life years, with more proximal prevention-related coverage indicators, such as screening and vaccination coverage. These indicators differ in causal distance from healthcare delivery and in the extent to which they are directly controllable by health systems. Therefore, the estimated frontier should be interpreted as a multidimensional benchmarking construct rather than as a single homogeneous production function. In addition, some outputs, particularly life expectancy and healthy life years, are influenced by broader social, behavioural, and environmental determinants beyond healthcare delivery. Fourth, the DEA model used unrestricted endogenous weights. This flexibility allows each country to be evaluated under its most favourable output mix, but it may also allow some outputs to receive low or zero implicit weight for specific countries. Because DEA multiplier weights can be non-unique across optimal solutions and sensitive to model specification, they were not interpreted as substantive policy weights or reported as primary results. Future research could apply weight restrictions or assurance-region models to examine whether the findings are robust to minimum-weight constraints on core health indicators. Fifth, the sample size was necessarily limited to countries with complete data, and some variables referred to the nearest available year rather than exactly 2022, which may have introduced residual comparability problems and limited discriminatory power. Sixth, although bootstrap correction improves inference, DEA remains a relative and model-dependent measure of efficiency rather than an absolute measure of performance. Finally, the second-stage ecological analysis was conducted on a small number of countries, which limited statistical power; it should therefore be interpreted as exploratory rather than strongly explanatory.

## 5. Conclusions

Improving health system performance in Europe cannot be reduced to increasing expenditure alone. From a public health perspective, the priority is to strengthen the capacity of health systems to convert available resources into measurable population health gains, preventive coverage, and healthy longevity. This requires not only adequate financing, but also better allocation of resources, stronger workforce planning, and greater emphasis on prevention and early detection as core functions of health systems rather than residual components of care. These findings support the use of cross-country benchmarking to inform health policy, identify resource configurations associated with higher relative inefficiency under the specified model, and promote more value-oriented planning. For policymakers, the main implication is that reforms should focus on governance, integration, and public health capacity, with the aim of improving outcomes before simply expanding spending. Future research should build on this approach using broader indicators of quality, unmet need, and disease burden in order to support more comprehensive and policy-relevant assessments of health system performance.

## Figures and Tables

**Table 1:** Baseline output-oriented CRS DEA scores across 25 European countries.

Country	Output-Oriented DEA Score, ϕ (Model 1)	Technically Efficient
Croatia	1.000	Yes
Czechia	1.000	Yes
Estonia	1.000	Yes
Greece	1.000	Yes
Hungary	1.000	Yes
Latvia	1.000	Yes
Lithuania	1.000	Yes
Poland	1.000	Yes
Slovak Republic	1.042	No
Slovenia	1.122	No
Spain	1.166	No
Portugal	1.230	No
Finland	1.231	No
Sweden	1.284	No
Italy	1.380	No
Denmark	1.526	No
Norway	1.652	No
Luxembourg	1.720	No
Netherlands	1.787	No
United Kingdom	1.976	No
France	2.167	No
Ireland	2.219	No
Belgium	2.230	No
Austria	2.283	No
Germany	2.617	No

Note: Scores are reported to three decimal places. In the output-oriented formulation, ϕ=1.000 indicates frontier status, whereas ϕ>1.000 indicates the proportional output expansion required to reach the estimated frontier. Scores below 1 are not possible under this convention; the reciprocal 1/ϕ corresponds to a conventional 0–1 efficiency scale.

**Table 2:** Robustness output-oriented DEA scores after inclusion of physician density.

Country	Output-Oriented DEA Score, ϕ (Model 2, Physician-Adjusted)	Technically Efficient
Croatia	1.000	Yes
Czechia	1.000	Yes
Estonia	1.000	Yes
Finland	1.000	Yes
France	1.000	Yes
Greece	1.000	Yes
Hungary	1.000	Yes
Latvia	1.000	Yes
Lithuania	1.000	Yes
Luxembourg	1.000	Yes
Poland	1.000	Yes
Slovenia	1.000	Yes
United Kingdom	1.007	No
Belgium	1.011	No
Slovak Republic	1.027	No
Sweden	1.058	No
Denmark	1.129	No
Italy	1.138	No
Spain	1.143	No
Netherlands	1.186	No
Ireland	1.222	No
Portugal	1.230	No
Norway	1.309	No
Germany	1.430	No
Austria	1.598	No

Note: Scores are reported to three decimal places. In the output-oriented formulation, ϕ=1.000 indicates frontier status, whereas ϕ>1.000 indicates the proportional output expansion required to reach the estimated frontier.

**Table 3:** Bootstrap bias-corrected output-oriented CRS DEA scores and 95% confidence intervals, ordered by bias-corrected score.

Country	Original Output-Oriented DEA Score, ϕ (Model 1)	Bias-Corrected Output-Oriented DEA Score, ϕ (Model 3)	95% CI Lower	95% CI Upper
Lithuania	1.000	1.100	1.016	1.200
Poland	1.000	1.114	1.010	1.232
Czechia	1.000	1.141	1.012	1.262
Hungary	1.000	1.173	1.016	1.294
Slovak Republic	1.042	1.183	1.054	1.334
Estonia	1.000	1.192	1.009	1.333
Latvia	1.000	1.207	1.015	1.369
Croatia	1.000	1.211	1.014	1.433
Slovenia	1.122	1.219	1.129	1.350
Greece	1.000	1.234	1.020	1.405
Spain	1.166	1.273	1.171	1.404
Finland	1.231	1.377	1.246	1.515
Portugal	1.230	1.381	1.242	1.529
Sweden	1.284	1.432	1.303	1.598
Italy	1.380	1.562	1.395	1.737
Denmark	1.526	1.717	1.544	1.933
Norway	1.652	1.840	1.669	2.061
Netherlands	1.787	1.916	1.798	2.131
Luxembourg	1.720	1.976	1.743	2.327
United Kingdom	1.976	2.291	1.996	2.706
France	2.167	2.410	2.199	2.646
Ireland	2.219	2.437	2.248	2.709
Belgium	2.230	2.507	2.259	2.744
Austria	2.283	2.590	2.310	2.878
Germany	2.617	2.941	2.652	3.299

Note: Bias-corrected scores are reported on the same output-oriented scale as the original DEA scores. Values greater than 1 indicate greater distance from the estimated frontier.

**Table 4:** Second-stage Simar–Wilson truncated regression of bootstrap bias-corrected DEA inefficiency scores.

Variable	Coefficient	Bootstrap SE	95% CI Lower	95% CI Upper
Intercept	−9.422	7.772	−26.220	3.822
Log GDP per capita (PPS)	1.993	1.023	0.219	4.197
Population aged ≥65 years (%)	0.121	0.123	−0.096	0.382
Adult obesity prevalence (%)	−0.043	0.035	−0.112	0.025
Current tobacco use prevalence (%)	−0.008	0.038	−0.076	0.072

## Data Availability

https://databank.worldbank.org/source/world-development-indicators (accessed on 13 January 2026). https://data.un.org/Data.aspx?d=PopDiv&f=variableID%3A77 (accessed on 27 February 2026). https://ec.europa.eu/eurostat/databrowser/view/hlth_hlye/default/table?lang=en (accessed on 11 April 2026). https://immunizationdata.who.int/global/wiise-detail-page/poliomyelitis-vaccination-coverage (accessed on 19 December 2025). https://view.officeapps.live.com/op/view.aspx?src=https%3A%2F%2Fstat.link%2Ffiles%2F918d8db3-en%2Feji4wd.xlsx&wdOrigin=BROWSELINK (accessed on 15 January 2026). https://data-explorer.oecd.org/vis?tm=breast&pg=0&snb=10&df[ds]=dsDisseminateFinalDMZ&df[id]=DSD_HEALTH_PROC%40DF_SCREEN&df[ag]=OECD.ELS.HD&df[vs]=1.1&dq=................CICDMNBR.&pd=2010,&to[TIME_PERIOD]=false (accessed on 3 March 2026). https://ec.europa.eu/eurostat/databrowser/view/TEC00114/default/table (accessed on 17 February 2026). https://data360.worldbank.org/en/indicator/WB_HCP_OBESITY (accessed on 4 March 2026).
